# Social Support and Meaning of Life in Women with Breast Cancer

**DOI:** 10.4314/ejhs.v32i4.6

**Published:** 2022-07

**Authors:** Ali Jadidi, Farzad Ameri

**Affiliations:** 1 Ph.D in Nursing, School of Nursing, Arak University of Medical Sciences, Arak, Iran; 2 Bs in Nursing, School of Nursing, Arak University of Medical Sciences, Arak, Iran

**Keywords:** Breast cancer, Family, Mental health, Social support, Women

## Abstract

**Background:**

Social support is known as an affection-oriented coping mechanism when a person is involved with cancer. Therefore, this study was conducted to investigate the relationship between family social support and the meaning of life in women with breast cancer.

**Methods:**

In this cross-sectional study, 84 women with a mean age of 60 (SD = 5.7) years with breast cancer who were admitted to a teaching hospital participated. Data were collected using social support and meaning of life questionnaires. After collecting the completed questionnaires and entering the data into the computer, the analysis was performed using SPSS software and using t-test, ANOVA, and Pearson correlation test at a significant level of 0.01.

**Results:**

The mean score of their social support was 39.35 ± 9.51, respectively and the meaning of life was 29.5 ± 7.49. ANOVA results indicated that the social support score and meaning of life had no significant relationship with any of the demographic variables. Also, the findings suggest that there is a statistically significant correlation between social support and the meaning of life (r = 0.773, P < 0.001).

**Conclusion:**

It is proposed to increase the level of social support from the family to help improve the meaning of life in cancer patients.

## Introduction

Cancer is potentially a life-threatening illness that causes significant disorders for patients in all aspects of personal, family, and social life (1). According to a study conducted in the United States in 2015, cancer is now regarded as the second death factor in that area, and it is expected to surpass heart disease as a major cause of death in the next few years. Breast cancer is one of the major causes of mortality and morbidity (2, 3) which allocates 15% of cancer-related deaths among women (4). In the United States, breast cancer has the largest number of new cases in the female population (5). In Iran, breast cancer with a prevalence of 23% is the most common cancer among females and it is regarded as 87% of the total cause of death in the age group of 15–49 years and 99% of the total cause of death in the age group of 50–69 years in 2019 (6).

In western countries, breast cancer is mainly observed in ages over 50, while based on some studies conducted in Iran in 2018, the number of breast cancer patients aged between 40 and 49 is higher than in other age groups. In addition, the number of younger patients in Iran is higher than that of the Western countries (7). Social support as one of the most emotional coping mechanisms is recognized as the greatest and most powerful coping force for patients to cope with cancer and its stressful conditions successfully and effectively (8,9). Social support is defined as “the perception or experience of a person from “social protection” namely, he should feel he is cared for, valued, and made a part of a social network with contributions and commitments (10).

Purposefulness in life and high social support is associated with a significant reduction in the level of the disease symptoms (11). Fear of death, cancer recurrence, community rejection, abandonment, isolation, defamation, and disability are regarded as some of the greatest concerns of cancer patients (12,13). Psychological support can lead to significant improvements in distress caused by a cancer diagnosis (14). Breast cancer patients who have instrumental support can pursue medical and therapeutic treatment, observe diet and have mobility with motivation, which may improve the survival of the patient and protect the individual against inability (15). Emotional-social support is usually provided by a confidential person who may be able to reduce the patient's stress (16). The support from others acts as a shield against the negative outcomes of illness and treatment and consequently, makes a strong correlation with the patient's psychological function (17). Cancer increases the need for social support due to the many changes which occur during life (8).

Cancer is known as a social phenomenon due to its association with social isolation and death (18). According to some new studies, existential and spiritual issues are useful in relieving and protecting cancer patients, because the supportive care concepts have transcended the focus on pain control and physical symptoms, and in general, have highlighted the development of existential and spiritual issues such as the meaning of life, hope, and spirituality (19,20). The results of some studies indicated that the meaning of life is an essential requirement in the search for the fulfillment of meaning in life and refers to the purposeful life (21–23).

The meaning of life should be equivalent to the purpose of life, and the goals of life should be valued positively (24). Other researchers regarded the meaning of life as a goal and coherence in life, or as the personal significance of certain conditions of life (25,26). Curtius reported that the meaning of life refers to believing in a purposeful pattern of the world, which, in turn, can be derived from religion or spirituality (27). Others have the same ideas, for instance, the closest definition of the meaning of life is “Understanding the pattern, the social class, coherence and having a purpose, pursuit of worthy goals, and having a sense of accomplishment of the goals in individuals” (28). In another study, Jadidi demonstrated that the meaning of life is realized in helping each other, having a goal, maintaining family relationships, and living well (29). Having meaning or purpose in life has a positive relationship with psychological factors and leads to adaptation, life satisfaction, good psychological feeling, and social support (30). Therefore, it seems that social support results in reducing cancer patients and changing their meaning of life. Therefore, the present study was conducted to investigate the relationship between social support resources and the meaning of life among women with breast cancer.

## Materials and Methods

Based on an analytic cross-sectional study, 84 women with breast cancer who were referred to an educational hospital in Arak city (Iran) were selected. The data collection instrument was a self-report questionnaire. The inclusion criteria were the person's awareness, the time and place of patients, the absence of other cancers and chronic diseases such as diabetes, deafness, blindness, Persian speaking, lack of mental illness, not using psychotropic drugs, lack of addiction, and completion of treatment with one of the methods of radiotherapy, surgery, and chemotherapy.

The scale of the meaning of life was presented by Stiger, Fraser, Oishi, and Coler in 2006 to assess the existence or and attempt to find the meaning. The questionnaire consisted of ten questions and its reliability was estimated at 86% for the life assessment and 87% for the subscale of meaning (28,29). Further, the social support questionnaire (Family Scale) of Peresidano and Heller was used. The reliability of this questionnaire was estimated at 82% (31). Its options are (Yes, No, I do not know). The score of the “I do not know” option is always zero, while it is +1 for questions 3, 4, 16, 19, and 20, and the score of the option of “Yes” for the rest of the questions was + 1. The total score range of questions ranged between 0 and 20. From the respondents' perspective, a high score means more social support. Moreover, the social support questionnaire with an alpha coefficient of 0.90 had dramatic internal coordination. Validity-based data was based on the 20 main questions of scales before separating family support. The final alpha for the questionnaire ranged between 0.88 and 0.91. The questionnaire had good concurrent validity. The demographic questionnaire included age, sex, marital status, stage of cancer, economic status, duration of diagnosis, and type of treatment.

**Statistical methods**: The collected data were analyzed by *t*-test, ANOVA, and Pearson correlation coefficient at a significant level of 0.01.

**Ethical considerations**: The researcher began the study after receiving an ethics code under No: IR.ARAKMU.REC.1395.190 and the introduction letter for the interview issued by the Research Ethics Committee of Arak University of Medical Sciences. Regarding the physical and psychological conditions of cancer patients, the questionnaire was only given to those who were in a suitable condition and tended to participate in the study. Moreover, they were informed about the objectives and confidentiality of the obtained data and there was no need to mention their names in the questionnaire.

**Patient consent statement**: All of the participants were ensured of information confidentiality and written consent was obtained from them for participation in the study.

## Results

The sample included 84 women with breast cancer and all of them were married and had a child. The age of diagnosis in most patients (47.6%) ranged between 35 and 40. Although more than 92% had health insurance, most (61.9%) said they did not have enough income to treat their illness. Other demographic characteristics of the subjects are presented in Table 1.

**Table 1:** Characteristics of Participants, (N=84)

Variables		Mean(SD)
Age		60(5.7)
		**Frequency(Percent)**
Residence style	City	49 (58.3)
	Village	35(41.7)
Educational level	Illiterate	65(77.4)
	Elementary	10(11.9)
	Guidance	2(2.4)
	High school	4(4.8)
	University	3(3.6)
Occupation	Housewife	77(91.7)
	Employee	7(8.3)
Stage of disease	Stage1	51(60.7)
	Stage2	21(25)
	Stage3	12(14.3)

Table 2 indicates the mean and standard deviation of the main variables of the study. ANOVA results indicated that none of these variables have a statistically significant relationship with age, educational level, and stage of the disease. However, social support and meaning of life are significantly associated with the age of diagnosis so the score of these variables between the ages of 35 and 40 was higher than that of other ages. Furthermore, based on t-test results, none of these variables have a statistically significant relationship with location, insurance, and income.

**Table 2:** Social Support and Meaning of Life of Participants, (N=84)

Variables	Min	Max	Mean(SD)
Social support	25.00	77.00	39.34(9.51)
Search for the meaning	5.00	23.00	14.52(5.1)
Existence of meaning in life	9.00	22.00	14.97(2.87)
Meaning of life	16.00	43.00	29.50(7.49)

In addition, findings indicated that social support had a positive correlation with the mean of life meaning and two subscales (P=0.000). The Pearson correlation coefficient between social support variables and meaning of life was r=0.773. Namely, more than 59% of the changes in these two variables have the same variance. In other words, 59% of the changes in the meaning of life are related to social support and vice versa (Table 3).

**Table 3:** Correlations between Social Support and Meaning of Life (N=84)

Variable		Search for the meaning	Existence of meaning in life	Meaning of life
**Social support**	P-value	.000	.000	.000
	R	.784	.622	.773

## Discussion

This study aimed to investigate the relationship between social support from the family and the meaning of life among women with breast cancer in Arak (Iran). The results showed that the mean score of patients' social support from the family is moderate. The finding is consistent with the results of other studies such as the study of French et al. on the social support of MS patients, the study of Chang on social support by children's mothers, as well as the study of Carreno et al. on women with genital cancer, which indicated that social support for subjects is moderate (31–33).

The results also showed that there was no significant relationship between social support and all demographic variables, except the place of residence. This finding is not in line with the results of other studies. For example, Lv et al. and Leung et al. in their study demonstrated that social support is related to income level (34,35). In the same vein, the study of Horwood et al. indicated that social support had a significant relationship with educational level as people with higher educational levels had a better understanding of themselves, illness, people around them, and their support (36). Furthermore, the meaning of life and its two sub-scales were not related to any of the demographic variables.

The results of this study suggest that social support provided by a close relative may influence the patient's meaning of life in an inpatient care setting. Both of these indicators are known to be important during hospitalization and receiving routine care. A similar conclusion was reached in the research done by Blau et al. who found a significant correlation by exploring the relationship between the degree of social support satisfaction and the meaning of life (37). Similarly, a study by Seo and Jeong shows that the meaning of life is significantly correlated with social support from the family (38).

Experiencing one's life as meaningful is positively influenced by the amount of value others place on a person and the amount of support they receive from others for people dealing with incurable conditions. In the meantime, social support provided by a close relative has a greater and deeper influence on the patient's meaning of life and overall life satisfaction. This is even more important in cancer patients; Because they have difficulty understanding the meaning of life. Therefore, trying to maintain and promote the meaning of life is of particular importance in these patients (39,40). So these dimensions should be considered and discussed in the design of interdisciplinary treatment protocols during hospitalization.

This study indicated that the level of social support from the family is related to understanding the meaning of life among women with cancer. Prevention and intervention efforts need to be directed toward improving social support, family support in particular, and assisting patients in finding meaning in life after a diagnosis of cancer.

Palliative caregivers can alleviate patient suffering by supporting the patients' efforts in rebuilding the meaning of their lives. Interventions designed to improve the patient's sense of life purpose, efficacy, and self-worthiness can effectively serve more than one goal. Members of the palliative care team should establish a good relationship with the patient and explore the mysteries associated with the patient's meaning of life and with the patient's adaptation to their situation.

Considering that some subjects were illiterate, or did not have the patience to study and answer the questions, some trained scholars were used to question the subjects and fill in the questionnaire. Thus, attempts were made to ask questions in a simple language so that low-level educated patients can answer the questions correctly. Therefore, there may be a bias in answering the questionnaire questions.
